# Case of Rupture of the Heart

**Published:** 1852-01

**Authors:** Edwin R. Maxson


ART. V.—*Case of Rupture of the Heart.* Reported by EDWIN R. MAXSON, M. D.

In March of 1849, Mrs. K——, a lady of thirty, of common health, while carrying her child of two years old, playfully lifted it over her head, when she felt an uneasiness in the cardiac region. This was soon followed by a sensation of warmth, and an unusual fluttering of the heart, which, however, partially subsided in a little time.

She now kept about the house most of the time for the following two days, though she was somewhat indisposed from a feeling of warmth and fullness, or pressure, about the heart, with occasional irregular action of that organ. The preceding symptoms were followed, toward the close of the second day, by slight syncope, dizziness, and a general feeling of uneasiness and prostration. On retiring to rest, at evening, she became thirsty and called for drink, and the next moment she was dead.

I was invited, by the politeness of two medical gentlemen, to assist in making an examination; which we did forty-eight hours after death. We found the pericardium filled with coagulated blood, making an envelope, nearly covering the heart. There was a rupture of the right auricle sufficiently large to admit a crow quill, the edges being loose, thin, and somewhat ragged.

